# Complexities in the estimation of overdiagnosis in breast cancer screening

**DOI:** 10.1038/sj.bjc.6604638

**Published:** 2008-09-02

**Authors:** S W Duffy, E Lynge, H Jonsson, S Ayyaz, A H Olsen

**Affiliations:** 1 Cancer Research UK Centre for Epidemiology Mathematics and Statistics, Wolfson Institute of Preventive Medicine, Charterhouse Square, London EC1 M 6BQ, UK; 2 Institute of Public Health, University of Copenhagen, Øster Farimagsgade 5, opg. B, Copenhagen K DK-1014, Denmark; 3 Department of Radiation Sciences, Oncology, Umeå University, Umeå, Sweden

**Keywords:** mammography, breast cancer screening, overdiagnosis

## Abstract

There is interest in estimating and attributing temporal changes in incidence of breast cancer in relation to the initiation of screening programmes, in particular to estimation of overdiagnosis of breast cancer as a result of screening. In this paper, we show how screening introduces complexities of analysis and interpretation of incidence data. For example, lead time brings forward time- and age-related increases in incidence. In addition, risk factors such as hormone replacement therapy use have been changing contemporaneously with the introduction of screening. Although we do not indicate exactly how such complexities should be corrected for, we use some simple informal adjustments to show how they may account for a substantial proportion of increased incidence, which might otherwise erroneously have been attributed to overdiagnosis. We illustrate this using an example of analysis of breast cancer incidence data from Sweden.

In the 1990s, many countries experienced substantial increases in breast cancer incidence (Chu *et al*, 1996; Botha *et al*, 2003), whereas since 2002, it has fallen in the USA (Ravdin *et al*, 2007), both being subjects of the study (Glass *et al*, 2007). The recent decline was mostly attributed to a lower prevalence of hormone replacement therapy (HRT) use (Ravdin *et al*, 2007), whereas the levelling off of screening mammography rates in the late 1990s has been suggested as contributing (Glass *et al*, 2007). In relation to increased incidence in the 1990s, this is usually attributed to the introduction of mammographic screening (Jonsson *et al*, 2005; Seppänen *et al*, 2006). It is well established that this causes an immediate rise in incidence, mainly due to the early diagnosis of a many prevalent asymptomatic cancers (Jonsson *et al*, 2005; Svendsen *et al*, 2006). Also important is overdiagnosis, that is, the detection by screening of cancer which would never have been diagnosed in the host's lifetime if screening had not taken place (Olsen *et al*, 2006; Paci *et al*, 2006; Biesheuvel *et al*, 2007), estimated rates of overdiagnosis, ranging from around 3 to 50% or more (Biesheuvel *et al*, 2007).

In estimating overdiagnosis either in primary research or in reviews, the complexities in interpreting cancer incidence data in the epoch of screening are important (Paci and Duffy, 2005). For example, one of the present authors (Jonsson *et al*, 2005) identified an excess incidence in the screening epoch in Sweden, which remains unexplained, but specifically did not attribute this to overdiagnosis. Indeed, the authors cited a number of other influences, including the prevalence screen effect mentioned above and potential changes in risk-factor prevalence, notably the use of HRT. Nevertheless, certain studies have interpreted the above results as direct estimates of overdiagnosis (Biesheuvel *et al*, 2007; Glass *et al*, 2007).

This potential overinterpretation of incidence patterns is symptomatic of the difficulty in estimating overdiagnosis in the context of disease screening. Before screening was initiated, breast cancer incidence was increasing in many countries (Ravdin *et al*, 2007). However, the complex interaction among increasing incidence, lead time, age at diagnosis and calendar period of diagnosis is often poorly appreciated (Paci and Duffy, 2005). Here, we describe the complexities of the incidence/screening relationship and illustrate their potential to explain substantial increases in incidence, using one study as an illustration (Jonsson *et al*, 2005).

## Materials and methods

### Screening and incidence

In the first instance, it should be noted that before the introduction of screening, breast cancer incidence was rising in many parts of the world (Pisani, 1992) so that any estimate of excess incidence associated with screening should take such trends into account (Jonsson *et al*, 2005). In addition, changes in risk-factor prevalences contemporaneous with the advent of screening may add to underlying incidence beyond the prescreening time trend. A prime example of this is the two- to threefold increase in HRT use in Sweden between the early 1980s and the early 1990s (Søgaard *et al*, 2000). As we shall see below, the interrelationship between time trends and the effect of screening can be complicated.

When a screening programme is introduced, and all members of a target population are offered screening for the first time, a major rise in incidence is observed. The randomised trials show that around 3 years of incidence is harvested at the first screen in women aged 50 or above (Tabar *et al*, 1992). Thus, if it takes 2 years to carry out the prevalence screen, around a 100% increase in incidence will be observed during the period. This is an important component of the evidence for the estimated duration of the preclinical screen-detectable period, which is typically 2–4 years (Paci and Duffy, 1991; Tabar *et al*, 1992). This is largely not overdiagnosis, but early diagnosis, as is evidenced by the fact that incidence of interval cancers in the screened population thereafter is lower than the expected total incidence in the absence of screening (Tabar *et al*, 1992). The interpretation (Biesheuvel *et al*, 2007) of one reported prevalence screen effect (Jonsson *et al*, 2005) as ‘overdetection’ (Biesheuvel *et al*, 2007) indicates that this is still not fully appreciated.

What is even less well appreciated is the fact that a lead-time effect persists beyond the introductory phase of screening. Even if there were no overdiagnosis at all, the incidence would not return to, or fall below, that expected in the absence of screening until after the cohort screened has passed beyond the upper age limit for screening. This is due to several factors, but three of them are particularly often neglected:

1. *Prevalence screening of women reaching the lower age limit of the screening programme.* Seven years or more after the start of screening in Sweden, a 54% increase of breast cancer incidence was observed at ages 50–59 (Jonsson *et al*, 2005). Several Swedish counties, however, started screening at age 50, so this will not be purely incidence screening, and there will also be prevalence screening of women aged 50–51, a point raised in this paper.
2. *Increased age-specific incidence due to lead time.* If screening confers an average lead time of 3 years on screen-detected cancers and two-thirds of cancers diagnosed in the programme are screen detected, the average lead time in the programme will be 2 years. This means that age 52 incidence will be observed at age 50, age 53 incidence at age 51 and so on. As incidence increases with age, this will artificially increase the observed age-specific incidence.
3. *Increased incidence due to anticipation of the temporal trend.* As noted above, in almost all countries where mammographic screening is common, breast cancer incidence was increasing over time before the programme started. With a 2-year average lead time, we will observe 1995 incidence in 1993, 1996 incidence in 1994 and so on, again artificially inflating the observed incidence.

**Figure 1:**
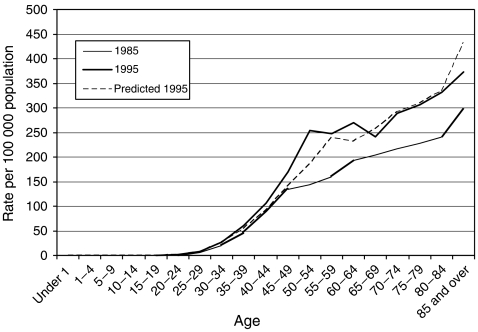
Age-specific breast cancer incidence in the UK in 1985, before the screening programme, and 1995, during the screening programme, with expected incidence in 1995 calculated from incidence trends observed before the breast screening programme began.

Figure 1 shows the incidence of breast cancer in England and Wales in 1985 and 1995, plus a third line representing the expected incidence in 1995 based on incidence trends in 1980–86, before screening was started. The first intuitive interpretation of such a graph is to interpret the vertical differences as increased incidence. However, in the age range covered by screening, 50–64, it is instructive to consider the horizontal differences. These will be a product of the three lead-time phenomena noted above, together with genuine underlying changes in risk-factor prevalence which may have caused an increased incidence with time, and overdiagnosis. The important point to note is that estimation of overdiagnosis should take into account the lead-time phenomena in addition to overdiagnosis and changes in incidence.

### Example – breast cancer incidence in Sweden, ages 50–69

In the study of incidence in eleven counties in Sweden, with a target population for screening of 463 405 women, it was estimated that 7 years or more after the initiation of screening at ages 50–59, the breast cancer incidence relative to that expected from prescreening trends was 1.69, a 69% excess (Jonsson *et al*, 2005). After a lead-time adjustment involving moving some cases in the age group 40–49 to the 50–59 group and some from the 50–59 to the 60–69 age group, the relative incidence was 1.54.

## Results

To consider the possible contribution to the raw figure of 1.69 of some of the complexities referred to above, we first remove the effects of the prevalence screening of those reaching age 50. In the prevalent phase, the excess incidence was 84%. The counties starting screening at age 50 comprised around 26% of the population, and within these counties, 20% of the screens at ages 50–59 were estimated to be prevalence screens. If *x* denotes the increased incidence excluding prevalence screens, we have: 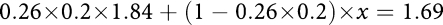 This solves to give *x*=1.68, a very minor change.

Next, with respect to the effect of lead time on age-specific incidence, incidence in the absence of screening increases by around 64% between age groups 50–59 and 60–69 (Jonsson *et al*, 2005). A 2.4-year lead time as estimated in that study for this age group therefore corresponds to a 13% increase in incidence. Dividing 1.68 by 1.13 gives a figure of 1.49. This method implicitly replaces the incidence in the age group 50–59 by that in the group 2.4 years older than this, without physically shifting cases between age groups in the analysis.

To consider an adjustment for use of HRT, this increased the female population as a whole from around 1.2% to around 4.0% between 1985 and 1995 (Søgaard *et al*, 2000). If we assume that around half of HRT use occurred in the 13% of the population aged 50–59, this would imply an increase from 4.6 to 15.3% in the period. Using the estimated relative risk of 1.66 with current HRT use from the million women study (Beral *et al*, 2003), and a method for aggregate exposure measures (Duffy *et al*, 2007), this would mean a 7% increase in risk due to HRT; dividing the 1.49 by 1.07 gives a relative incidence of 1.39, a 39% excess.

## Discussion

We have described some of the complexities of interpretation and analysis in estimation of overdiagnosis in breast cancer screening, and illustrated these with an example from Sweden (Jonsson *et al*, 2005). The 39% excess is considerably smaller than the 54% estimated by the lead-time adjustment of simply shifting of a number of cases by age group. It is still a rough approximation, and we do not claim that it is the correct estimate of overdiagnosis. It relies on a number of assumptions, and is based on invasive cancer incidence alone: inclusion of ductal carcinoma *in situ* might give larger estimates of excess incidence. It does, however, illustrate that adjustment for some of the complexities in incidence by time and age and in screening-induced lead time can account for a substantial proportion of any observed excess incidence coincident with screening, before any consideration of overdiagnosis is necessary. It also suggests that the results of the Swedish study have been overinterpreted as overdiagnosis estimates by others (Biesheuvel *et al*, 2007; Glass *et al*, 2007). Thus, there are also reasons for believing that much of the 69% uncorrected excess observed at ages 50–59 in the 7 years post-screening epoch is largely due to other factors than overdiagnosis. Also, after the prevalence period, the incidence fell dramatically, as might be expected, but then equally dramatically rose again to the prevalence level over the following 6 years (Jonsson *et al*, 2005). It is difficult to see how overdiagnosis rates should fall and then rise again in this manner.

The fact that lead time and related phenomena are likely to continue to inflate the observed incidence until after the cohort has ceased to be screened for some time implies that cumulative incidence in screened or invited cohorts compared to unscreened or uninvited is a desirable source of data for estimation of overdiagnosis. Using cumulative incidence in the Malmö Trial study and control groups, followed up until after screening had ceased in the study group (Zackrisson *et al*, 2006), overdiagnosis rates of 7–8% were estimated, considerably more plausible than observational incidence estimates which did not take full account of the extent of current and previous screening exposure in the cohorts studied (Zahl *et al*, 2004). There are problems of interpretation of Zackrisson's results, notably the relatively recent cessation of screening in one age group, but the results are certainly more consistent with the disease progression modelling which explicitly takes lead time into account (Olsen *et al*, 2006).

Finally, we note that overdiagnosis in this context is an epidemiological rather than a pathological concept, the definition of disease concerning what would not have been diagnosed if the screening had not taken place. It therefore is not necessarily restricted to *in situ* or ‘minimal’ invasive cancer, although intuitively one would expect that the majority of overdiagnosed cases would be in one of these two categories. Definitive estimation of overdiagnosis of invasive and *in situ* carcinoma in breast screening remains to be done. This paper emphasises that any such definitive estimation needs to take into account the complexities of the relationship between screening lead time and patterns of incidence by time and age. It is likely that after adjusting for these, overdiagnosis estimates will be smaller than many rates quoted in the past. The challenge for the future will be the avoidance of overtreatment of screen-detected and early stage cancers.
